# Chemical Proteomic Analysis of Serine Hydrolase Activity in Niemann-Pick Type C Mouse Brain

**DOI:** 10.3389/fnins.2018.00440

**Published:** 2018-07-03

**Authors:** Eva J. van Rooden, Annelot C. M. van Esbroeck, Marc P. Baggelaar, Hui Deng, Bogdan I. Florea, André R. A. Marques, Roelof Ottenhoff, Rolf G. Boot, Herman S. Overkleeft, Johannes M. F. G. Aerts, Mario van der Stelt

**Affiliations:** ^1^Molecular Physiology, Leiden Institute of Chemistry, Leiden University, Leiden, Netherlands; ^2^Bioorganic Synthesis, Leiden Institute of Chemistry, Leiden University, Leiden, Netherlands; ^3^Institute of Biochemistry, Christian-Albrechts-Universität zu Kiel, Kiel, Germany; ^4^Department of Medical Biochemistry, Academic Medical Center, University of Amsterdam, Amsterdam, Netherlands; ^5^Medical Biochemistry, Leiden Institute of Chemistry, Leiden University, Leiden, Netherlands

**Keywords:** chemical proteomics, activity-based protein profiling, endocannabinoid system, hydrolases, Niemann-Pick type C

## Abstract

The endocannabinoid system (ECS) is considered to be an endogenous protective system in various neurodegenerative diseases. Niemann-Pick type C (NPC) is a neurodegenerative disease in which the role of the ECS has not been studied yet. Most of the endocannabinoid enzymes are serine hydrolases, which can be studied using activity-based protein profiling (ABPP). Here, we report the serine hydrolase activity in brain proteomes of a NPC mouse model as measured by ABPP. Two ABPP methods are used: a gel-based method and a chemical proteomics method. The activities of the following endocannabinoid enzymes were quantified: diacylglycerol lipase (DAGL) α, α/β-hydrolase domain-containing protein 4, α/β-hydrolase domain-containing protein 6, α/β-hydrolase domain-containing protein 12, fatty acid amide hydrolase, and monoacylglycerol lipase. Using the gel-based method, two bands were observed for DAGL α. Only the upper band corresponding to this enzyme was significantly decreased in the NPC mouse model. Chemical proteomics showed that three lysosomal serine hydrolase activities (retinoid-inducible serine carboxypeptidase, cathepsin A, and palmitoyl-protein thioesterase 1) were increased in Niemann-Pick C1 protein knockout mouse brain compared to wild-type brain, whereas no difference in endocannabinoid hydrolase activity was observed. We conclude that these targets might be interesting therapeutic targets for future validation studies.

## Introduction

**FIGURE 1:**
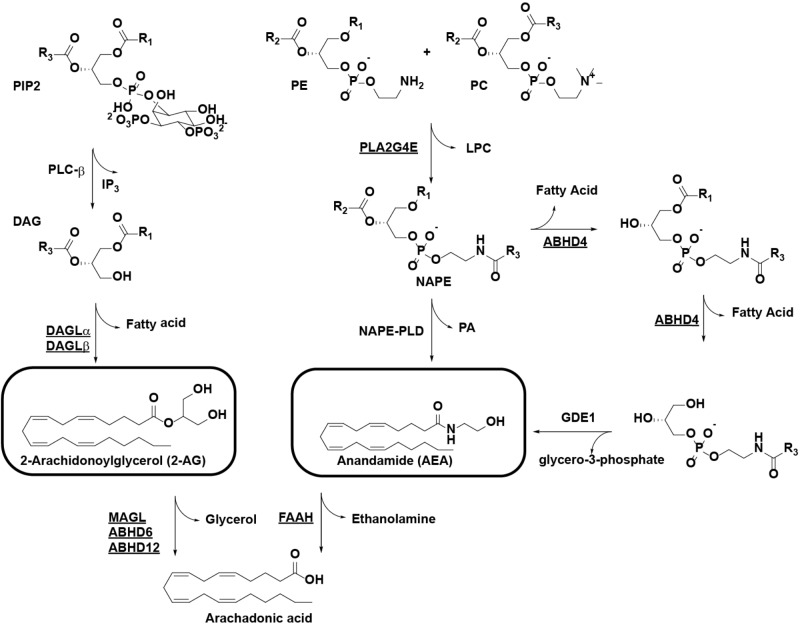
Pathways for the biosynthesis and degradation of the endocannabinoids 2-AG and AEA. Enzymes with known activity-based probes are underlined. R1/R2, alkyl chains, R3, arachidonoyl. ABHD, α/β-hydrolase domain-containing protein; AEA, anandamide; 2-AG: 2-arachidonoylglycerol; DAG, diacylglycerol; DAGL, diacylglycerol lipase; FAAH, fatty acid amide hydrolase; GDE1, glycerophosphodiesterase 1; LPC, lysophosphatidylcholine; MAGL, monoacylglycerol lipase; NAPE, *N*-acylphosphatidylethanolamine; PC, phosphatidylcholine; PE, phosphatidylethanolamine; PIP2, phosphatidylinositol 4,5-bisphosphate; PLA2G4E, phospholipase A2 group 4E; PLC, phospholipase C; PLD, phospholipase D.

The endocannabinoid system (ECS) consists of the cannabinoid type 1 and 2 receptors (CB_1_R and CB_2_R) and their endogenous ligands: the endocannabinoids. The lipids anandamide (AEA) and 2-arachidonoylglycerol (2-AG) are the best characterized endocannabinoids (Di Marzo et al., 2004). The enzymes responsible for endocannabinoid biosynthesis and degradation are also part of the ECS, with most endocannabinoid degrading enzymes belonging to the serine hydrolase family (**Figure 1**; Blankman and Cravatt, 2013). The combined activities of biosynthetic enzymes and degradative enzymes tightly regulate endocannabinoid concentrations in the brain. For example, 2-AG is mainly produced from 1-acyl-2-AG by the diacylglycerol lipases (DAGLs), DAGLα and DAGLβ, while it is degraded by monoacylglycerol lipase (MAGL) and to a minor extent by α,β-hydrolase domain-containing protein 6 (ABHD6) and α,β-hydrolase domain-containing protein 12 (ABHD12). Multiple pathways are known for the biosynthesis of AEA, but they all use *N*-acylphospatidylethanolamine (NAPE) as a central precursor, including the serine hydrolase α,β-hydrolase domain-containing protein 4 (ABHD4) and the β-metallolactamase *N*-acylphospatidylethanolamine-phospholipase D (NAPE-PLD). NAPE is synthesized by the calcium-dependent *N*-acyltransferase phospholipase A2 group 4E (PLA2G4E) (Ogura et al., 2016). Fatty acid amide hydrolase (FAAH) terminates AEA signaling by hydrolysis of its amide bond.

Endocannabinoid hydrolases that are part of the serine hydrolase superfamily can be studied using activity-based protein profiling (ABPP), a method which uses chemical probes for measuring enzyme activity in complex biological samples. Previously, we have used ABPP to compare serine hydrolase activity in wild-type and CB_1_R knockout mice (Baggelaar et al., 2017). Comparative ABPP has also been successfully applied in the identification of new drug targets (Nomura et al., 2010a,b). With ABPP, enzyme activities that are deregulated in a certain pathophysiological state can be identified and the relative amount of active enzyme copies as compared to wild-type situations quantified. The classical fluorophosphonate (FP)-based probes (FP-TAMRA and FP-biotin; Liu et al., 1999) act as broad-spectrum serine hydrolase probes that label the endocannabinoid enzymes FAAH, MAGL, PLA2G4E (Ogura et al., 2016), ABHD6, and ABHD4 (Liu et al., 1999; Simon and Cravatt, 2010), whereas the tailor-made tetrahydrolipstatin (THL)-based probes (MB064 and MB108) label both DAGL isoforms (alpha and beta) and ABHD4, ABHD6, and ABHD12 (Baggelaar et al., 2013, 2015, 2017; Ogasawara et al., 2016). Of note, the fluorescent probe DH379 (Ogasawara et al., 2016) selectively targets DAGL and ABHD6 (Supplementary Figure S1).

Evaluation of endocannabinoid hydrolase activity in native tissue may provide insight in the role of the ECS in physiological and disease processes. Interestingly, endocannabinoid levels are elevated during neurodegeneration and neuroinflammation (van der Stelt and Di Marzo, 2005; Nomura et al., 2011; Chiurchiù et al., 2017). For example, endocannabinoid signaling is perturbed in various animal models of neurodegenerative diseases, including stroke (Hillard, 2008), traumatic brain injury (Schurman and Lichtman, 2017), Alzheimer’s disease (Bisogno and Di Marzo, 2008), Huntington’s disease (Maccarrone et al., 2007), Parkinson’s disease (Di Marzo et al., 2000; Maccarrone et al., 2003), and multiple sclerosis (Baker et al., 2000). It is hypothesized that regulation of ECS activity may provide therapeutic benefit for these types of neurological diseases (Scotter et al., 2010).

Niemann-Pick type C (NPC) is a neurodegenerative lysosomal storage disorder, which is associated with mutations in either of the genes encoding Niemann-Pick C1 protein (NPC1) or NPC2 (Vanier and Millat, 2003). Both genes encode lysosomal proteins that are sequentially involved in cholesterol transport out of the lysosomes *via* a so far unknown mechanism. Defects in the function of the soluble NPC2 or the lysosomal membrane protein NPC1 leads to primary accumulation of cholesterol and secondary storage of sphingomyelin, sphingosine, and glycosphingolipids in lysosomes of multiple cell types, thereby leading to visceral complications such as enlarged liver and spleen combined with progressive neurological disease (Vanier and Millat, 2003).

A NPC mouse model is available. These NPC mice have previously been studied using ABPP with a retaining β-glucosidase probe (Marques et al., 2015). This study showed increased activity of the non-lysosomal glucosylceramidase (GBA2) in NPC1 knockout mice (and consistent increased abundance of the protein by Western blot). Importantly, pharmacological inhibition of GBA2 ameliorated the neuropathology of these mice (Marques et al., 2015). Miglustat is approved as a drug, and initially thought to work through substrate reduction by inhibiting glucosylceramide synthase (Nietupski et al., 2012). However, as we have shown before, the molecular mechanism does not involve glucosylceramide synthase, and we hypothesized that the therapeutic effect seems at least partly due to off-target inhibition of Miglustat on GBA2 (Marques et al., 2015). It has been suggested that accumulation of sterols in lysosomes impaired in NPC1 (or NPC2) causes a more general lysosome dysfunction involving multiple hydrolases, such as lysosomal glucocerebrosidase (GBA; Ferraz et al., 2016). Additionally, mutations in NPC1 or NPC2 genes result in severe progressive neurodegeneration. These observations led us to hypothesize that the hydrolases of the ECS might play a role in this disease. There is no treatment available for NPC patients. Additionally, there is no information available about the status of the ECS in Niemann-Pick. Therefore, we set out to measure endocannabinoid hydrolase activity in the NPC mouse model using ABPP.

## Materials and Methods

### Animals

*Npc1^-/-^* mice, along with wild-type littermates (*Npc1^+/+^*), were generated as published previously (Marques et al., 2015). Briefly, the heterozygous BALB/c Nctr-*Npc1*^m1N^/J mice (stock number 003092) were obtained from The Jackson Laboratory (Bar Harbor, ME, United States). The mice used in the current study were crossed for six generations in c57/bl6 background. Mouse pups were genotyped according to published protocols (Loftus et al., 1997). The mice were housed at the Institute Animal Core Facility in a temperature- and humidity-controlled room with a 12-h light/dark cycle and given free access to food and water *ad libitum*. All animal protocols were approved by the Institutional Animal Welfare Committee of the Academic Medical Centre Amsterdam in Netherlands. At the age of 69 days, animals were first anesthetized with a dose of Hypnorm (0.315 mg/mL fenyl citrate and 10 mg/mL fluanisone) and Dormicum (5 mg/mL midazolam) according to their weight. The given dose was 80 μL/10 g body weight. Blood was collected by a heart puncture followed by cervical dislocation. Brains were dissected, rinsed with phosphate-buffered saline (PBS), snap frozen in liquid N_2_, and stored at -80°C for biochemistry. Brain tissue was obtained from six female mice, three wildtype and three knockout.

### Preparation of Mouse Tissue Proteome

The mouse brains were cut in half with a scalpel through the midsection (sagittal plane). The mouse brain halves were slowly thawed on ice. The thawed mouse brain halves were dounce homogenized in 1.5-mL cold (4°C) lysis buffer (20 mM HEPES pH 7.2, 2 mM DTT, 1 mM MgCl_2_, 25 U/mL benzonase) and incubated for 15 min on ice. The suspension was centrifuged (2500 *g*, 3 min, 4°C) to remove debris. The supernatant was collected and transferred to an ultracentrifuge tube. The debris was resuspended in 0.25-mL lysis buffer and resubjected to centrifugation. The combined supernatants were collected and subjected to ultracentrifugation (100,000 *g*, 45 min, 4°C, Beckman Coulter, Type Ti70 rotor). This yielded the membrane fraction as a pellet and the cytosolic fraction in the supernatant. The supernatant was collected and the membrane fraction was suspended in 1.5-mL storage buffer (20 mM HEPES pH 7.2, 2 mM DTT). The total protein concentration was determined with Quick Start Bradford assay (Bio-Rad). Membranes and supernatant fractions were both diluted to either 1.0 or 2.0 mg/mL (for proteomics and gel-based ABPP, respectively) and were used directly or flash frozen in liquid nitrogen and stored in aliquots at -80°C until use.

### Activity-Based Protein Profiling

#### Gel-Based

Mouse brain cytosol or membrane fraction (2.0 mg/mL) was incubated with activity-based probe MB064 (250 nM), TAMRA-FP (500 nM), or DH379 (1 μM) (20 min, rt, 2.5% DMSO). For the competition experiments with inhibitor DH376, this step was preceded by incubation with 100 nM inhibitor (30 min, rt, 2.5% DMSO). For the competition experiments with probe MB108, this step was preceded by incubation with 10 μM probe (30 min, 37°C, 2.5% DMSO). Laemmli buffer was added to quench the protein activity and the mixture was allowed to stand at rt for at least 5 min before the samples were loaded and resolved on sodium dodecyl sulfate polyacrylamide gel electrophoresis (SDS-PAGE) gel (10% acrylamide), together with PageRuler Plus Prestained Protein Ladder (Thermo Scientific). The gels were scanned using a ChemiDoc (Bio-Rad, Cy3 channel: expose 180 s for MB064, 60 s for DH379/TAMRA-FP, Cy5 channel 10 s for marker). After fluorescent scanning, the gels were stained with a Coomassie staining solution [0.25% (w/v) Coomassie Brilliant Blue in 50% MeOH, 10% AcOH, 40% MilliQ (v/v/v)]. The gels were scanned after destaining with MilliQ. Both the fluorescence and Coomassie images were analyzed using Image Lab 5.2. The Coomassie gel is used to determine protein loading (automatic lane/band detection, background subtraction not enabled, select whole lane as band). The fluorescence bands are quantified using automatic lane/band detection with background subtraction enabled. Using the Coomassie-corrected gel fluorescence values, the average is calculated for WT and KO and a two-sided Student’s *t*-test is performed. The activities of proteins were relatively quantified by setting the average WT at 100%.

#### Western Blot

After the SDS-PAGE gel was resolved and imaged, proteins were transferred to 0.2-μm polyvinylidene difluoride membranes with a Trans-Blot Turbo^TM^ Transfer system (Bio-Rad). Membranes were washed with TBS (50 mM Tris, 150 mM NaCl) and blocked with 5% milk (w/v, Elk magere melkpoeder, FrieslandCampina) in TBST (50 mM Tris, 150 mM NaCl, 0.05% Tween 20) for 1 h at rt. Membranes were then incubated with the primary antibody anti-DAGLα (1:1000, Cell Signaling Technology, #13626) in 5% BSA (w/v) in TBST (o/n, 4°C), washed with TBST, incubated with matching secondary antibody HRP-coupled-goat-anti-rabbit (1:5000, Santa Cruz, sc2030) in 5% milk in TBST (1 h, rt) and washed with TBST and TBS. Imaging solution (10 mL Luminol, 100 μL ECL enhancer, 3 μL H_2_O_2_) was added to develop membranes and chemiluminescence was detected on the ChemiDoc (Bio-Rad) using standard chemiluminescence settings. The signal was normalized to Coomassie staining and quantified with Image Lab 5.2.

#### Proteomics

Mouse brain membrane or soluble proteome (245 μL, 1.0 mg/mL) was incubated with 5 μL 0.5 mM MB108 (10 μM) or FP-Biotin (10 μM) for 1 h at rt. The labeling reaction was quenched and excess probe was removed by chloroform/methanol precipitation: 250 μL MilliQ, 666 μL MeOH, 166 μL CHCl_3_, and 150 μL MilliQ added subsequently to each sample with a brief vortex after each addition. After centrifugation (4000 rpm, 10 min), the top and bottom layer surrounding the floating protein pellet was removed. 600 μL MeOH was added and the pellet was resuspended by sonication with a probe sonicator (10 s, 30% amplitude). After centrifugation (14,000 rpm, 5 min), the methanol was removed and the protein pellet was redissolved in 250 μL 6 M urea/25 mM ammonium bicarbonate and allowed to incubate for 15 min. 2.5 μL 1 M DTT was added and the mixture was heated to 65°C for 15 min. The sample was allowed to cool to rt (∼5 min) before 20 μL 0.5 M iodoacetamide was added and the sample was alkylated for 30 min in the dark. 70 μL 10% (wt/vol) SDS was added and the proteome was heated for 5 min at 65°C. The sample was diluted with 2 mL PBS. 50 μL of 50% slurry of Avidin-Agarose from egg white (Sigma-Aldrich) was washed with PBS and added to the proteome sample in 1 mL PBS. The beads were incubated with the proteome for 3 h, while rotating. The beads were isolated by centrifugation (2500 *g*, 2 min) and washed with 0.5% (wt/vol) SDS in PBS (1×) and PBS (3×). The proteins were digested overnight with 500 ng sequencing grade trypsin (Promega) in 250 μL on-bead digestion buffer (100 mM Tris pH 8, 100 mM NaCl, 1 mM CaCl_2_, 2% ACN) at 37°C with vigorous shaking. The pH was adjusted with formic acid to pH 3 and the beads were removed. The peptides were isotopically labeled by on-stage tip dimethyl labeling. Wild-type and knockout samples were differently labeled with isotopic dimethyl labeling and combined after labeling to allow comparison (wild-type light and knockout heavy).

#### On-Stage Tip Dimethyl Labeling

The stage tips were made by inserting C18 material in a 200-μL pipet tip. The step-wise procedure given in the **Table 1** was followed for stage tip desalting and dimethyl labeling. The solutions were eluted by centrifugal force and the constitutions of the reagents are given below. For label-free quantification samples, steps 6 and 7 are omitted.

**Table 1:** Step-wise on-stage tip dimethyl labeling procedure.

Step	Aim	Solution	Centrifugation
1	Conditioning	Methanol (50 μL)	2 min 600 g
2	Conditioning	Stage tip solution B (50 μL)	2 min 600 g
3	Conditioning	Stage tip solution A (50 μL)	2 min 600 g
4	Loading	Load samples on stage tips	2.5 min 800 g
5	Washing	Stage tip solution A (100 μL)	2.5 min 800 g
6	Dimethyl labeling (5×)	20/20/40/40/30 μL L or M reagent	5 min 400 g
7	Washing	Stage tip solution A (100 μL)	2.5 min 800 g
8	Elution	Stage tip solution B (100 μL)	2.5 min 800 g


Stage tip solution A is 0.5% (vol/vol) FA in H_2_O. Stage tip solution B is 0.5% (vol/vol) FA in 80% (vol/vol) ACN in H_2_O. Dimethyl labeling reagents: phosphate buffer (50 mM; pH 7.5) with NaBH_3_CN 0.03 M containing either 2% (vol/vol) CH_2_O (Light) or CD_2_O (Medium). After the final elution step, the desired heavy and light samples were combined and concentrated on a Speedvac to remove the ACN. The residue was reconstituted in 95/3/0.1 H_2_O/ACN/FA (vol/vol) before LC/MS analysis. Tryptic peptides were measured either on an Orbitrap or Synapt mass spectrometer.

#### Orbitrap

Tryptic peptides were analyzed on a Surveyor nanoLC system (Thermo) hyphenated to a LTQ-Orbitrap mass spectrometer (Thermo) as previously described. Briefly, emitter, trap and analytical column (C18, 120Å) were purchased from Nanoseparations (Nieuwkoop, Netherlands) and mobile phases (A: 0.1% formic acid/H_2_O, B: 0.1% formic acid/ACN) were made with ULC/MS grade solvents (Biosolve). General mass spectrometric conditions were: electrospray voltage of 1.8–2.5 kV, no sheath and auxiliary gas flow, capillary voltage 40 V, tube lens voltage 155 V, and ion transfer tube temperature 150°C. Polydimethylcyclosiloxane (*m*/*z* = 445.12002) and dioctyl phthalate ions (*m*/*z* = 391.28429) from the environment were used as lock mass. Some 10 μL of the samples was pressure loaded on the trap column for 5 min with a 10-μL/min flow and separated with a gradient of 35 min 5–30% B, 15 min 30–60% B, and 5 min A at a flow of 300 μL/min split to 250 nL/min by the LTQ divert valve. Full MS scans (300–2000 *m*/*z*) acquired at high mass resolution (60,000 at 400 *m*/*z*, maximum injection time 1000 ms, AGC 106) in the Orbitrap was followed by three MS/MS fragmentations in the LTQ linear ion trap (AGC 5 × 103, max inj time 120 ms) from the three most abundant ions. MS/MS settings were: collision gas pressure 1.3 mT, normalized collision energy 35%, ion selection threshold of 750 counts, activation *q* = 0.25, and activation time 30 ms. Ions of *z* < 2 or unassigned were not analyzed and fragmented precursor ions were measured twice within 10 s and were dynamically excluded for 60 s. Data analysis was performed using Maxquant with acetylation (protein N term) and oxidation (M) as variable modifications. The false discovery rate was set at 1%, and the peptides were screened against reviewed mouse proteome (Uniprot). Serine hydrolases that were identified in at least two repetitive experiments and for which at least one unique peptide and two peptides in total were identified were considered as valid quantifiable hits. For proteins identified by both probes, the normalized ratios from Maxquant were combined for further analysis. The binary logarithm of each ratio was compared to 0 with a Student’s *t*-test. The resulting *p* values were subjected to a Benjamini–Hochberg correction, setting the false discovery rate at 10% (*q* = 0.1). Briefly, the *p* values of all quantifiable hits were ordered from lowest to highest, and the Benjamini–Hochberg statistic was calculated as *q* × (position in the list) divided by the number of tests. Subsequently, the proteins for which the *p* value is smaller than the BH statistic are controlled for a FDR of *q* × 10%.

#### Synapt

The peptides were measured as described previously for the NanoACQUITY UPLC System coupled to SYNAPT G2-Si high definition mass spectrometer. A trap-elute protocol, where 1 μL of the digest is loaded on a trap column (C18 100Å, 5 μM, 180 μM × 20 mm, Waters) followed by elution and separation on the analytical column (HSS-T3 C18 1.8 μM, 75 μM × 250 mm, Waters). The sample is brought onto this column at a flow rate of 10 μL/min with 99.5% solvent A for 2 min before switching to the analytical column. Peptide separation is achieved using a multistep concave gradient based on the gradients used in Distler et al. (2016). The column is re-equilibrated to initial conditions after washing with 90% solvent B. The rear seals of the pump are flushed every 30 min with 10% (vol/vol) ACN. [Glu^1^]-fibrinopeptide B (GluFib) is used as a lock mass compound. The auxiliary pump of the LC system is used to deliver this peptide to the reference sprayer (0.2 μL/min). A UDMS^e^ method is set up as described in Distler et al. (2016). Briefly, the mass range is set from 50 to 2,000 Da with a scan time of 0.6 s in positive, resolution mode. The collision energy is set to 4 V in the trap cell for low energy MS mode. For the elevated energy scan, the transfer cell collision energy is ramped using drift time specific collision energies (Distler et al., 2014). The lock mass is sampled every 30 s. Raw data are processed in PLGS (v3.0.3) and ISOQuant v1.5.

## Results

**FIGURE 2:**
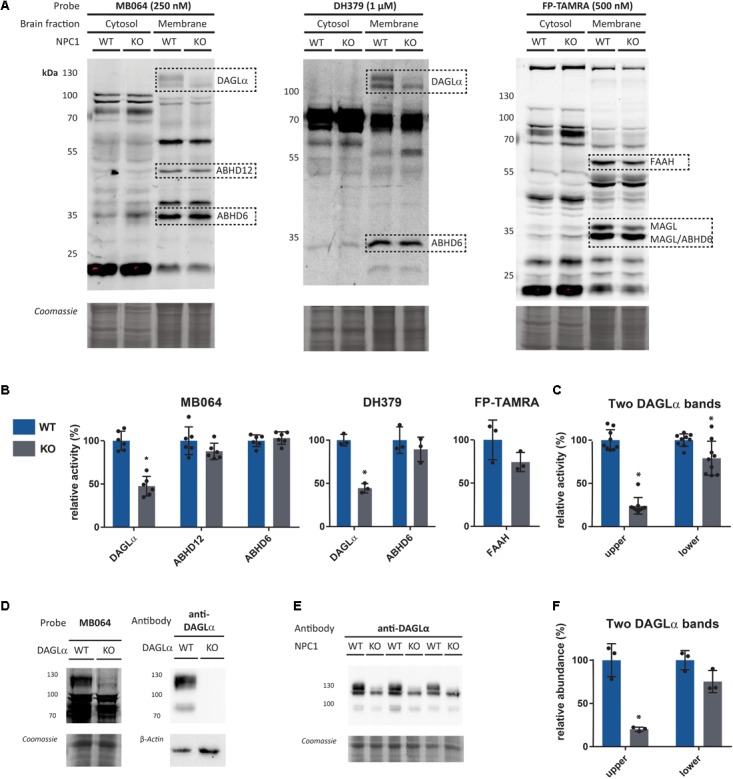
Gel-based *Npc1*^+/+^ (WT) vs. *Npc1^-/-^* (KO) comparison. **(A)** Enzyme activity in the brain fractions as measured by the probes MB064, DH379, and FP-TAMRA. **(B)** Quantification of relative enzyme activity in WT and KO for probe targets (average WT is set to 100%). *n* = 3 (mice), *n* = 2 (MB064), or *n* = 1 (DH379 and FP-TAMRA). **(C)** Quantification of probe labeling of the two observed bands for DAGLα as measured by MB064 and DH379 [gels: **(A)**]. Three mice per condition (*N* = 3) and three probe labeling experiments per lysate (*n* = 3). **(D)** DAGLα activity and abundance in DAGLα WT and KO mouse brain membrane proteome as measured by the probe MB064 and an anti-DAGLα antibody. **(E)** DAGLα abundance in NPC1 WT and KO mouse brain membrane proteome. **(F)** Quantification of antibody labeling of the two observed bands for DAGLα as measured by anti-DAGLα [blot: **(E)**]. Mean ± standard deviation is shown. ^∗^*p* < 0.05 (Student’s *t*-test).

In 95% of the patients afflicted by NPC, mutations in *NPC1* are observed. Therefore, the role of the ECS in this disease was investigated by comparison of endocannabinoid hydrolase activity between *Npc1*^+/+^ and *Npc1^-/-^* mouse brains. First, labeling profiles of MB064, DH379, and FP-TAMRA in wild-type and knockout mouse brains was evaluated (**Figure 2A**). Membrane and soluble fractions of both wild-type and knockout tissue were labeled with each probe separately, resolved on SDS-PAGE and visualized using in gel fluorescence scanning. Coomassie staining was used as a protein loading control. The fluorescence intensity of the bands corresponding to DAGLα (∼120 kDa), ABHD12 (∼50 kDa), ABHD6 (∼35 kDa), and FAAH (∼60 kDa) were quantified to determine the relative enzyme activity between knockout and wildtype (**Figure 2B**). Using the FP-TAMRA probe, the two bands corresponding to MAGL (∼35 kDa) were observed, but due to band overlap with ABHD6 these cannot be accurately quantified. Labeling of DAGLα significantly decreased in the knockout mice as compared to wildtype, while ABHD6 and ABHD12 activity was the same. FAAH labeling was slightly decreased in the knockout, but this decrease was not statistically significant. In the cytosolic fraction, an increase in intensity of a 75-kDa band as labeled by MB064 and FP-TAMRA was observed in the knockout brains. As reported previously (Gao et al., 2010; Baggelaar et al., 2017), DAGLα is identified as two separate bands. Remarkably, only the fluorescent band corresponding to the higher molecular weight was significantly decreased in the *Npc1^-/-^* mice as quantified by two separate probes MB064 and DH379 (**Figure 2C**). Both bands can be labeled with a DAGLα antibody and are absent in DAGLα KO mice (**Figure 2D**). To see if this observation was due to a decrease of protein abundance, a Western blot against DAGLα was performed (**Figure 2E**). The signal of antibody labeling was quantified (**Figure 2F**). The same pattern was observed for relative abundance as for relative activity: only the upper band corresponding to DAGLα is significantly decreased (**Figures 2C,F**).

**FIGURE 3:**
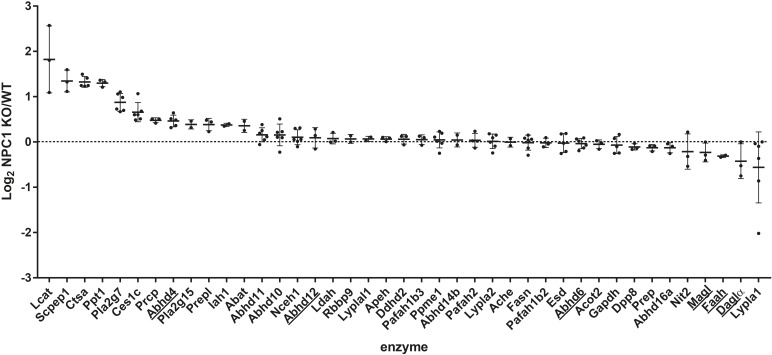
Chemical proteomics comparison of *Npc1*^+/+^ (WT) vs. *Npc1^-/-^* (KO). Log_2_ ratio of enzyme activity in *Npc1^-/-^* brain proteome compared to *Npc1*^+/+^. Activity was measured using FP-biotin or MB108 (both 10 μM). The data from both probes were combined for both the cytosolic and membrane fraction. The mean and standard deviation are shown. Endocannabinoid related enzymes are underlined. Statistical analysis by means of Student’s *t*-test (each ratio was compared to a log_2_ ratio of 0) and the resulting *p* values were subjected to Benjamini–Hochberg correction, setting the false discovery rate at 10% (^∗^ indicates significant difference).

Although several endocannabinoid enzymes can be quantified with gel-based ABPP, multiple bands remain unidentified (**Figure 2A**). Additional enzymes can be identified using a sensitive mass spectrometry-based method for ABPP (chemical proteomics). Therefore, to study a broader range of serine hydrolases and to confirm our observations from the gel-based assay, we also employed a chemical proteomics method using FP biotin and MB108 to assess the role of the ECS in NPC1. The enzymatic activity of 41 hydrolases in NPC1 knockout mouse brain were identified and compared with wild-type mouse brains (**Figure 3**). In line with the gel-based ABPP, we did not find any significant difference in the endocannabinoid hydrolase activity (ABHD12, ABHD6, FAAH, and MAGL) in NPC1 wild-type vs. knockout mice brain proteomes. The activity of endocannabinoid hydrolase ABHD4 is also unaltered in knockout compared to wildtype. Remarkably, and in contrast to the gel-based ABPP results, DAGLα activity in knockout mice brain proteomes was not decreased compared to wild-type mice. Of note, in a control experiment, biotinylated probe MB108 competes with both DAGLα bands labeled by the fluorescent probe MB064. Additionally, DAGL inhibitor DH376 did reduce DAGLα labeling in the chemical proteomics assay (Supplementary Figure S2). Finally, three hydrolases were significantly increased in knockout mouse brains compared to wild-type brains: retinoid-inducible serine carboxypeptidase (SCPEP1), cathepsin A (CTSA), and palmitoyl-protein thioesterase 1 (PPT1) (**Figure 3**).

## Discussion

We used ABPP to quantify the activity of the endocannabinoid hydrolases FAAH, ABHD6, ABHD12, ABHD4, and MAGL in NPC1 knockout mice and found that their activity is not affected in wild-type vs. NPC1 knockout mice. DAGLα activity seems to be decreased in the knockout mice based on the gel-based ABPP results. However, we found a discrepancy in the gel-based ABPP assay and the chemical proteomics assay in DAGLα activity between the NPC1 knockout mouse brains compared to wild-type mice. This discrepancy can possibly be caused by technical factors inherent to the two applied methodologies. In the gel-based ABPP assay, only the labeling of the upper DAGLα-band was abolished in the NPC1 knockout brain proteome. It could be that the peptides from the protein corresponding to the upper band from the wild-type mouse proteome are not detected in the mass spectrometer due to post-translational modifications, such as phosphorylation, acetylation, methylation, palmitoylation, or glycosylation. Peptides with such modifications are not found in our assay. If this would be true, a decrease in DAGLα activity in the NPC1 knockout mice would not be detected. Further experiments, such as direct quantification of 2-AG levels, are required to confirm a decrease in DAGLα activity in Niemann-Pick mice. In addition, it would be interesting to find the origin and role of the altered modification of DAGLα in NPC. If the gel-based ABPP results are confirmed, it would be worthwhile to measure 2-AG levels and test the hypothesis that decreased DAGL activity might represent a neuroprotective response by lowering the formation of pro-inflammatory prostaglandins (Ogasawara et al., 2016). Alternatively, it is known that 2-AG reduces cytotoxic edema in traumatic brain injury (Panikashvili et al., 2001), therefore boosting 2-AG brain levels by inhibition of MAGL may constitute a neuroprotective response.

Finally, we found that the activities of three non-endocannabinoid hydrolases, namely, CTSA, PPT1, and SCPEP1, were significantly elevated in NPC1 knockout mouse brains. These enzymes are lysosomal proteins, which is in line with the important role of NPC1 in lysosomes. SCPEP1 shows homology with CTSA (Kollmann et al., 2009) and a study with double knockout of SCPEP1 and CTSA suggests they share the same peptide substrate (Pan et al., 2014). Single knockout of SCPEP1 in mice resulted in viable mice without lysosomal impairment (Kollmann et al., 2009). Mutations in the CTSA gene cause galactosialidosis in humans (Galjart et al., 1990) and secondary deficiencies of beta-galactosidase and neuraminidase. This same phenotype is mirrored in a CTSA knockout mice mouse model (Korah et al., 2003). The protective role of the enzyme for galactosidase and neuraminidase is a structural function of the enzyme: mice with catalytic serine-to-alanine mutation have normal levels of β-galactosidase and neuraminidase (Seyrantepe et al., 2008). CTSA inhibitors have been tested in humans (Tillner et al., 2016), and CTSA is a therapeutic target for the treatment of cardiovascular diseases (Schreuder et al., 2014; Petrera et al., 2016). Thus, it would be interesting to test CTSA inhibitors in Niemann-Pick models. PPT1 is also a lysosomal enzyme (Lu et al., 2002) and the dysregulation of this enzyme causes a lysosomal storage and neurodegenerative disorder; infantile neuronal ceroid lipofuscinosis (Tillner et al., 2016). This enzyme removes palmitoyl modifications from proteins that are being degraded in the lysosome. In PPT1 knockout mice, cholesterol catabolism is altered (Ahtiainen et al., 2007). PPT1 has been proposed to hydrolyze 2-AG and might therefore be involved in the ECS (Wang et al., 2013). *In vivo* active inhibitors for PPT1 (Cognetta et al., 2015) have been identified and could therefore also be tested in Niemann-Pick models. To conclude, we have found no altered activity of endocannabinoid hydrolases in a NPC mouse model using our chemical proteomics assay. Three hydrolases were identified with upregulated activity in NPC1 knockout mice, which might be interesting therapeutic target for future studies.

## Data Availability Statement

The mass spectrometry proteomics datasets generated for this study have been deposited to the ProteomeXchange Consortium *via* the PRIDE (Vizcaíno et al., 2016) partner repository with the dataset identifier PXD008979.

## Author Contributions

EvR and AvE carried out the experiments and analyzed gel-based results. EvR analyzed the mass spectrometry data and supervised by MB. HD synthesized DH379 and supervised sample preparation. BF assisted with LC/MS measurements. AM and RO performed mice experiments and harvested tissue. EvR and MvdS wrote the manuscript, supported by RB and HO. MvdS and JA conceived the presented study. All authors provided critical feedback.

## Conflict of Interest Statement

The authors declare that the research was conducted in the absence of any commercial or financial relationships that could be construed as a potential conflict of interest.

## Abbreviations

ABHD: α/β-hydrolase domain-containing protein
ABPP: activity-based protein profiling
AEA: anandamide
2-AG: 2-arachidonoylglycerol
CB_x_R: cannabinoid type x receptor
CTSA: cathepsin A
DAGL: diacylglycerol lipase
ECS: endocannabinoid system
FAAH: fatty acid amide hydrolase
FP: fluorophosphonate
GBA2: non-lysosomal glucosylceramidase
MAGL: monoacylglycerol lipase
NAPE: *N*-acylphospatidylethanolamine
NPC: Niemann-Pick type C
PLA2G4E: phospholipase A2 group 4E
PLD: phospholipase D
PPT1: palmitoyl-protein thioesterase 1
SCPEP1: retinoid-inducible serine carboxypeptidase
SDS-PAGE: sodium dodecyl sulfate polyacrylamide gel electrophoresis
THL: tetrahydrolipstatin
