# Supplementary material for: Chemical Proteomic Analysis of Serine Hydrolase Activity in Niemann-Pick Type C Mouse Brain
Source: Front Neurosci. 2018 Jul 3;12:440. doi: 10.3389/fnins.2018.00440 (PMC6037894; doi:10.3389/fnins.2018.00440)
Supplement: Supplementary file 1 [file Image_1.PDF]

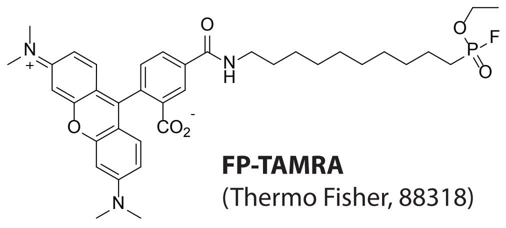

FP-TAMRA
(Thermo Fisher, 88318)

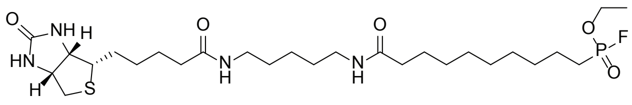

FP-biotin
(Santa Cruz, sc-215056A)

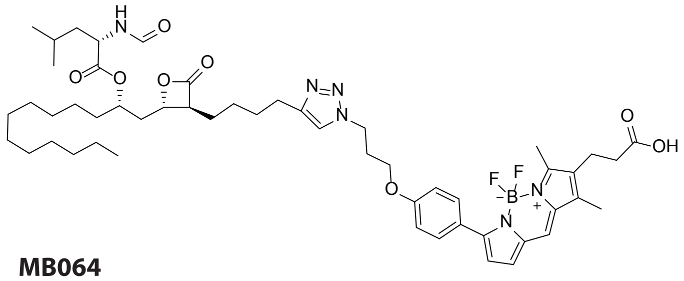

MB064
(Baggelaar, M. P. et al.
Angew. Chemie - Int. Ed. 52, 12081–12085, 2013)

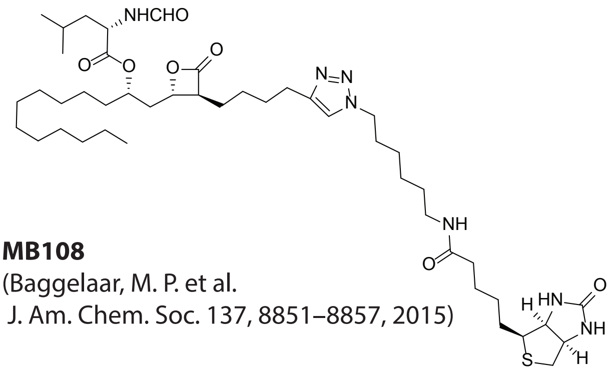

MB108
(Baggelaar, M. P. et al.
J. Am. Chem. Soc. 137, 8851–8857, 2015)

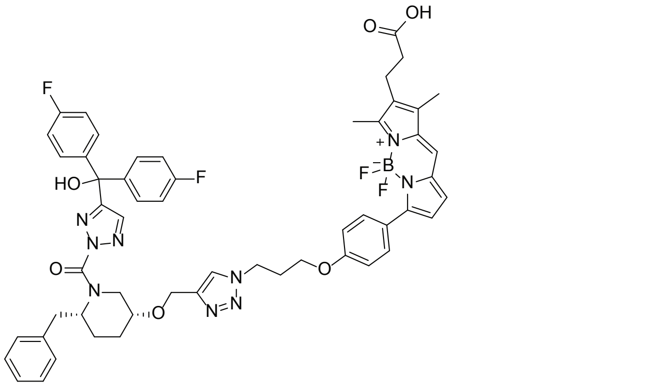

DH379
(Deng, H. et al.
Proc. Natl. Acad. Sci. 113, 26-33, 2016)

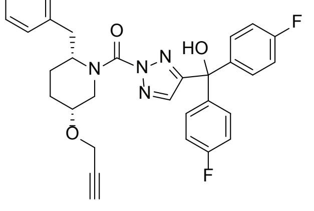

DH376
(Deng, H. et al.
Proc. Natl. Acad. Sci. 113, 26-33, 2016)

Supporting figure 1. Probes and inhibitors used in this study.

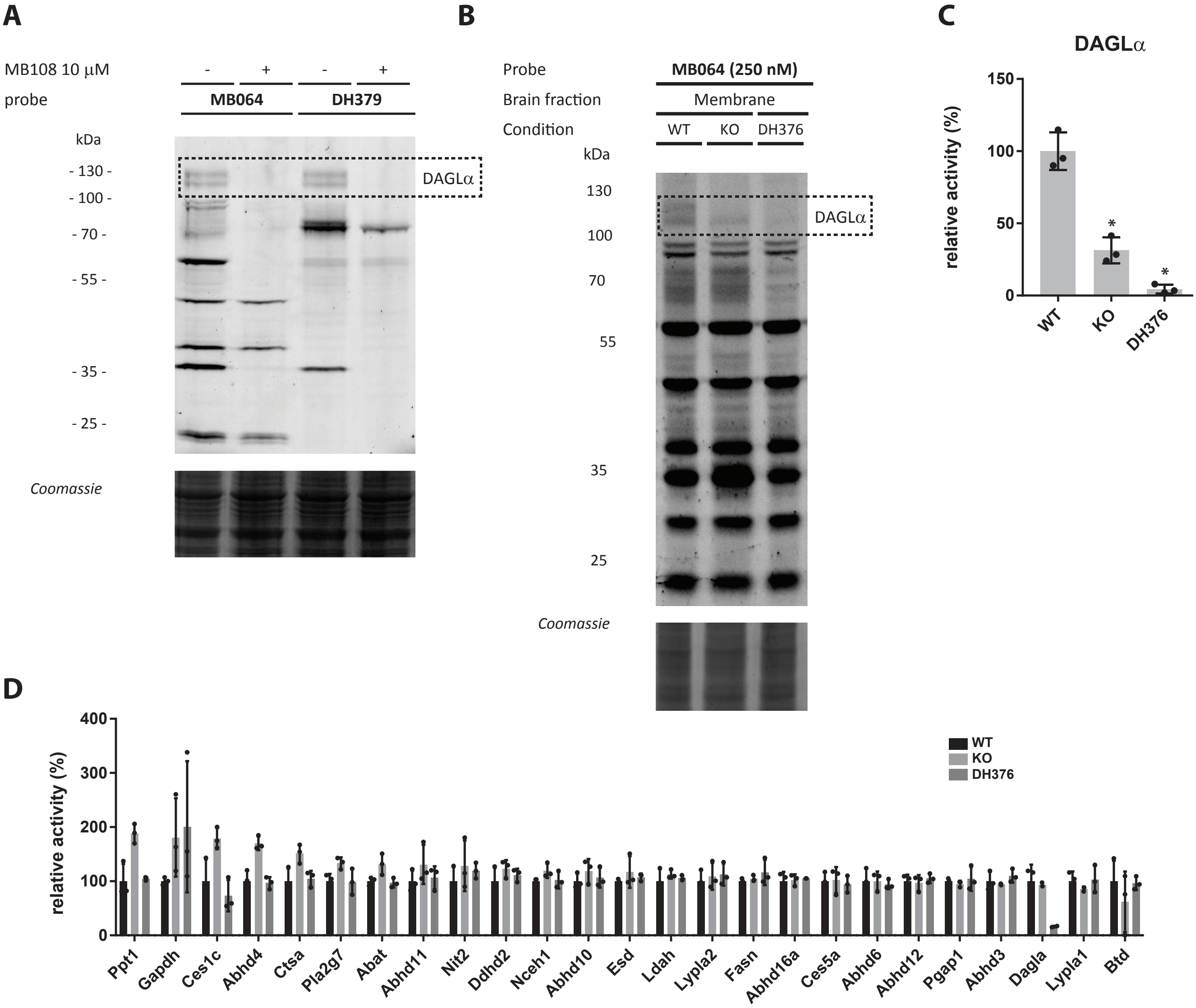

Supporting figure 2. Control experiments with MB108 and DAGLα inhibitor DH376 in mouse brain membrane proteome (A) Gel-based *Npc1* $^{+/+}$ (WT) pre-treatment with MB108 and labelling with MB064 and DH379. (B) Gel-based *Npc1* $^{+/+}$ (WT) vs *Npc1* $^{-/-}$ (KO) comparison and competition with inhibitor DH376. (C) Quantification of relative enzyme activity for DAGLα (average WT is set to 100%), N = 3. * $P < 0.05$ (Student's t-test). (D) Chemical proteomics comparison of *Npc1* $^{+/+}$ (WT) vs *Npc1* $^{-/-}$ (KO) and competition with inhibitor DH376. Activity was measured in the membrane fraction using MB108 (10 μM). Label-free quantification with IsoQuant. Statistical analysis by means of student's t-test (KO and inhibitor conditions compared to WT) and the resulting p-values were subjected to Benjamini-Hochberg correction, setting the false discovery rate at 10% (* indicates significant difference).

Full gels

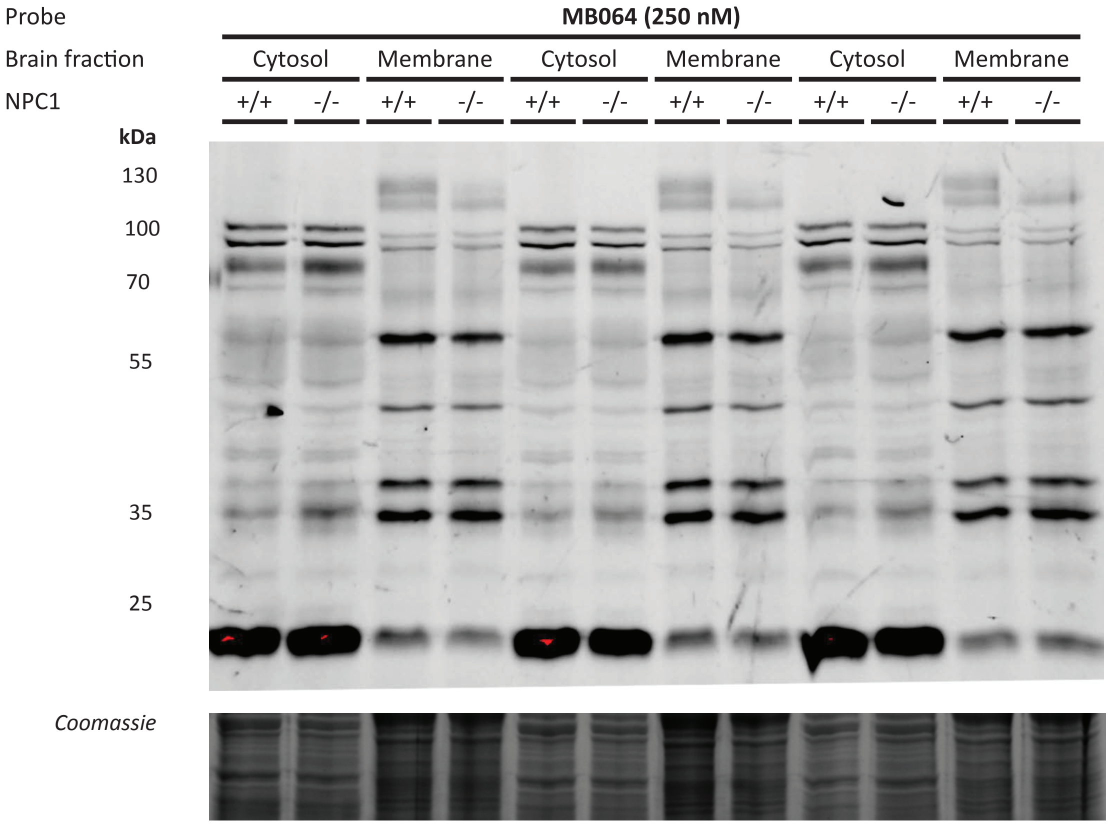
Probe
MB064 (250 nM)
Brain fraction
Cytosol
Membrane
Cytosol
Membrane
Cytosol
Membrane
NPC1
+/+
-/-
+/+
-/-
+/+
-/-
+/+
-/-
+/+
-/-
+/+
-/-
kDa
130
100
70
55
35
25
Coomassie

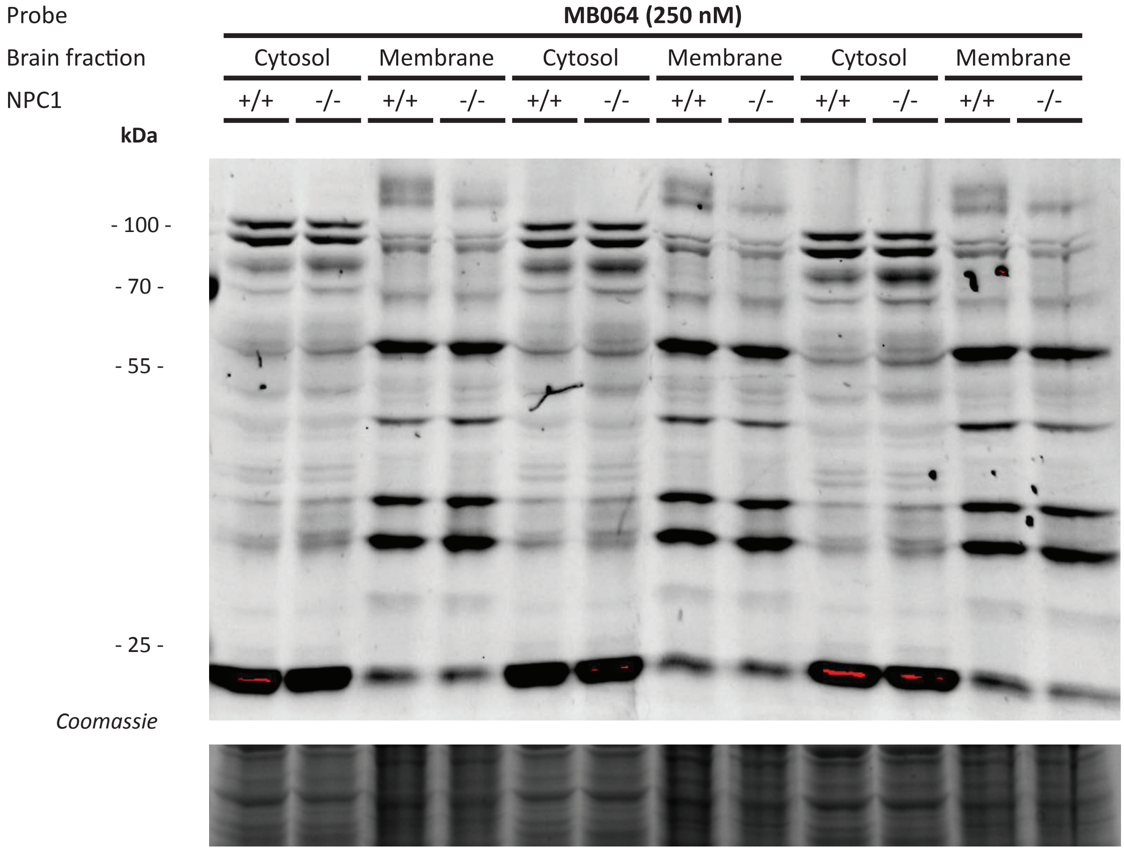
Probe
MB064 (250 nM)
Brain fraction
Cytosol
Membrane
Cytosol
Membrane
Cytosol
Membrane
NPC1
+/+
-/-
+/+
-/-
+/+
-/-
+/+
-/-
+/+
-/-
+/+
-/-
kDa
- 100 -
- 70 -
- 55 -
- 25 -
Coomassie

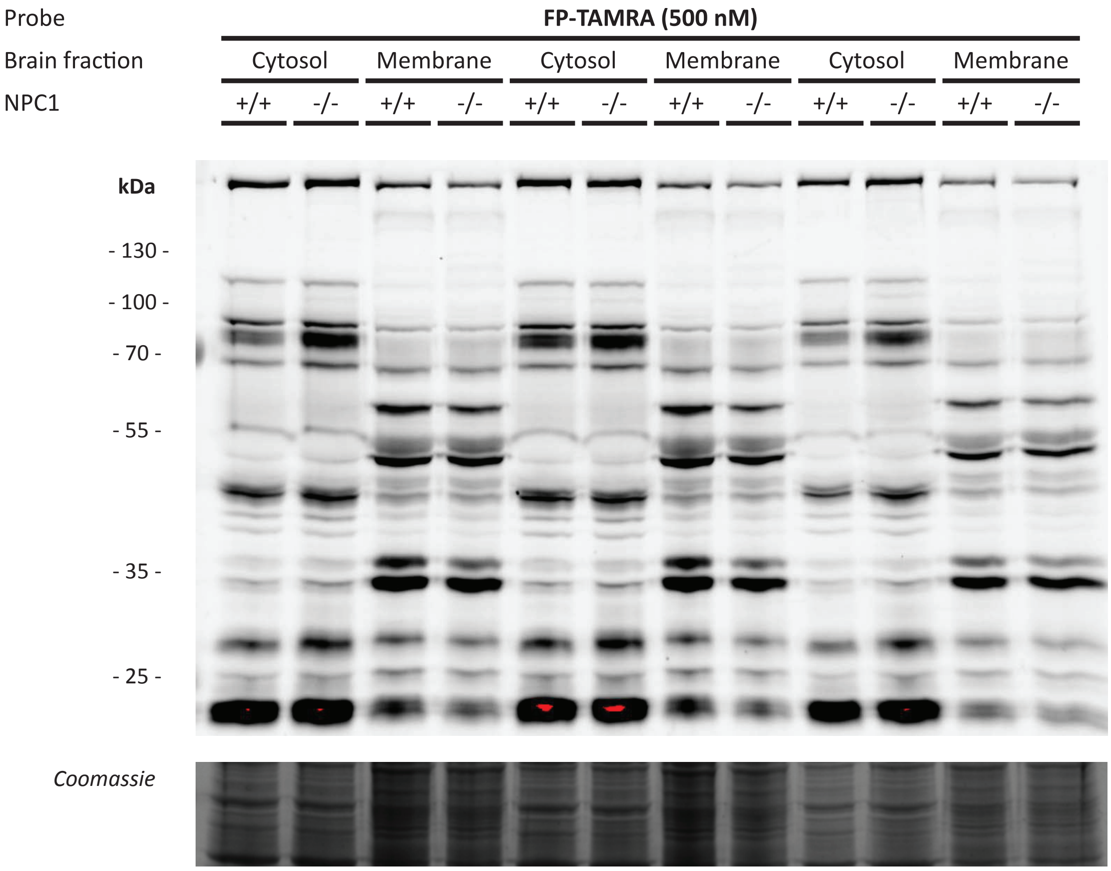

Probe
FP-TAMRA (500 nM)
Brain fraction
Cytosol
Membrane
Cytosol
Membrane
Cytosol
Membrane
NPC1
+/+
-/-
+/+
-/-
+/+
-/-
+/+
-/-
+/+
-/-
+/+
-/-
kDa
- 130 -
- 100 -
- 70 -
- 55 -
- 35 -
- 25 -
Coomassie

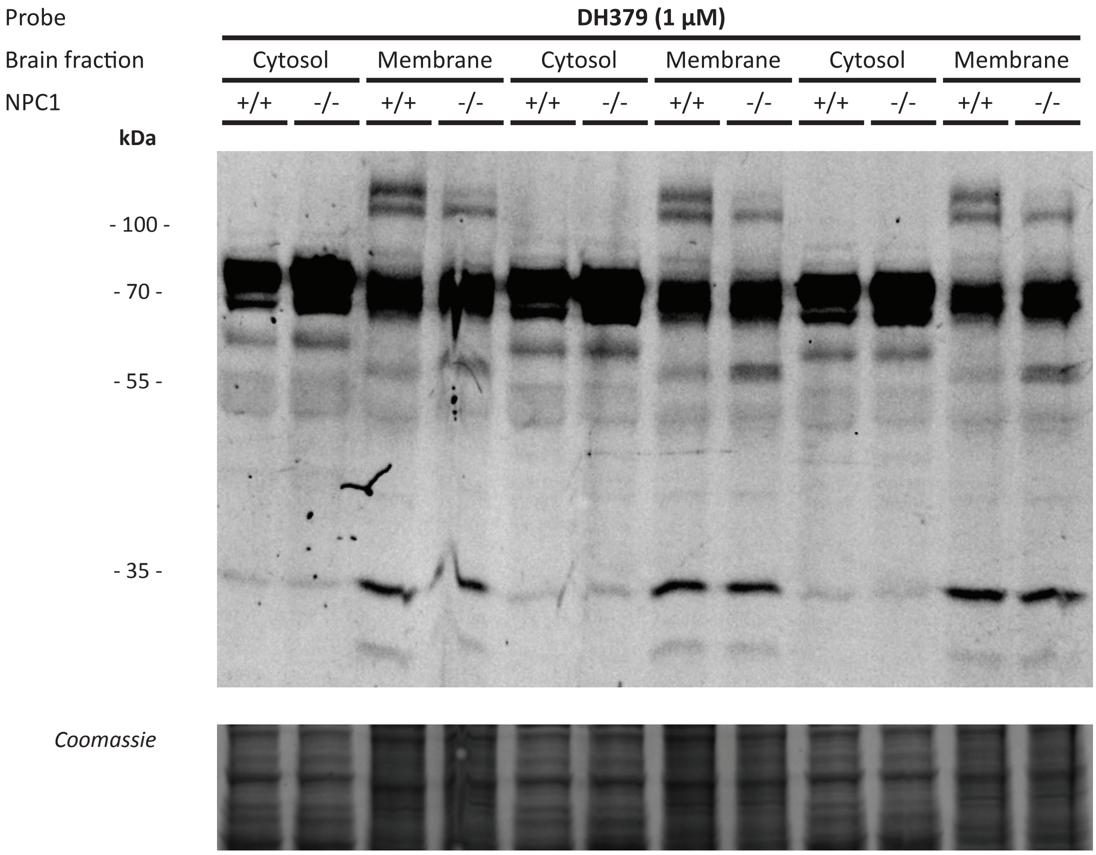

Probe
DH379 (1 μM)
Brain fraction
Cytosol
Membrane
Cytosol
Membrane
Cytosol
Membrane
NPC1
+/+
-/-
+/+
-/-
+/+
-/-
+/+
-/-
+/+
-/-
+/+
-/-
kDa
- 100 -
- 70 -
- 55 -
- 35 -
Coomassie

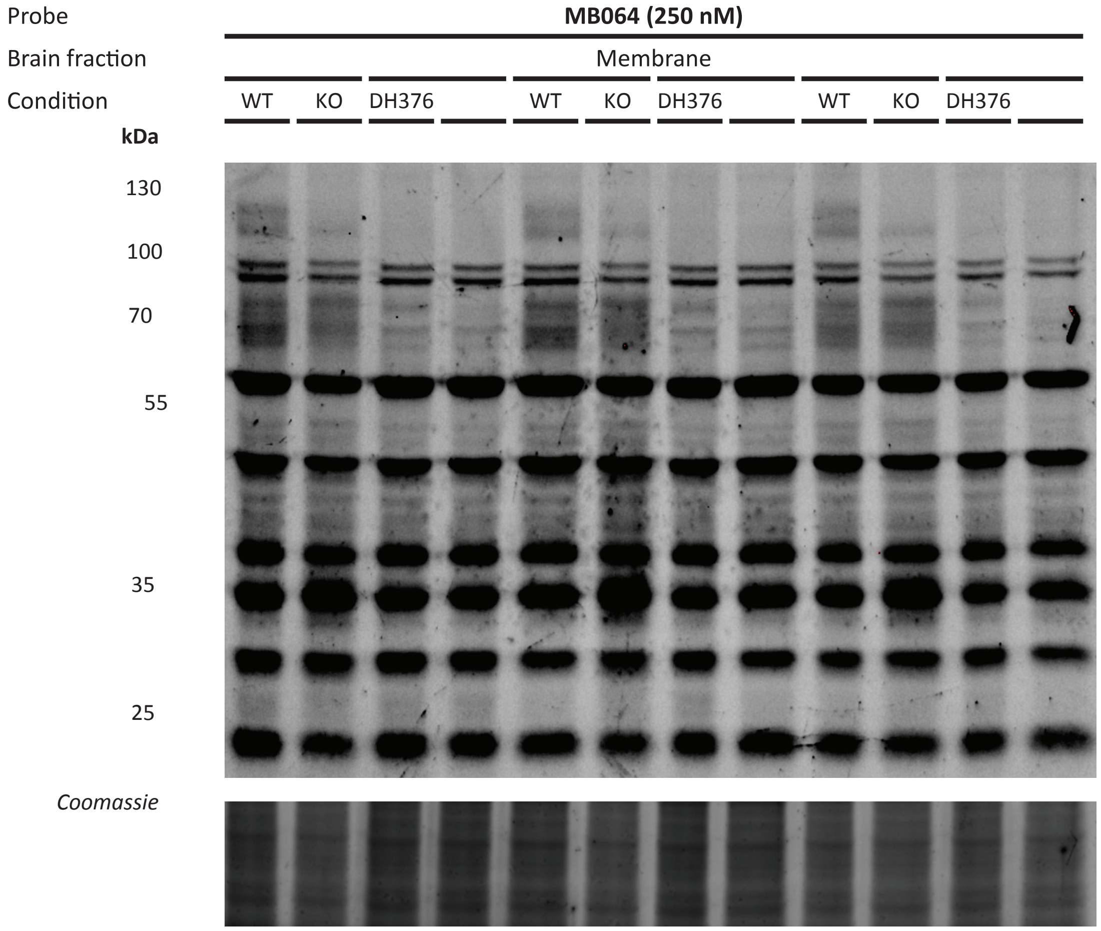
Probe
MB064 (250 nM)
Brain fraction
Membrane
Condition
WT
KO
DH376
WT
KO
DH376
WT
KO
DH376
kDa
130
100
70
55
35
25
Coomassie

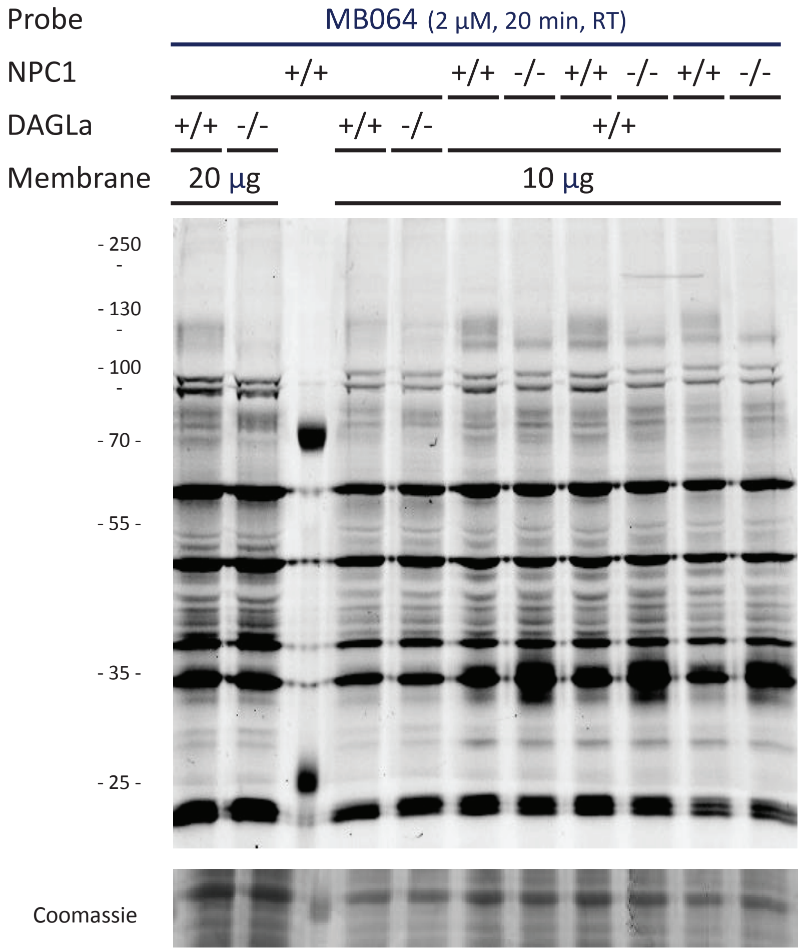
Probe
MB064 (2 μM, 20 min, RT)
NPC1
+/+
+/+ -/- +/+ -/- +/+ -/-
DAGLa
+/+ -/-
+/+ -/-
+/+
Membrane
20 μg
10 μg
- 250
- 130
- 100
- 70 -
- 55 -
- 35 -
- 25 -
Coomassie

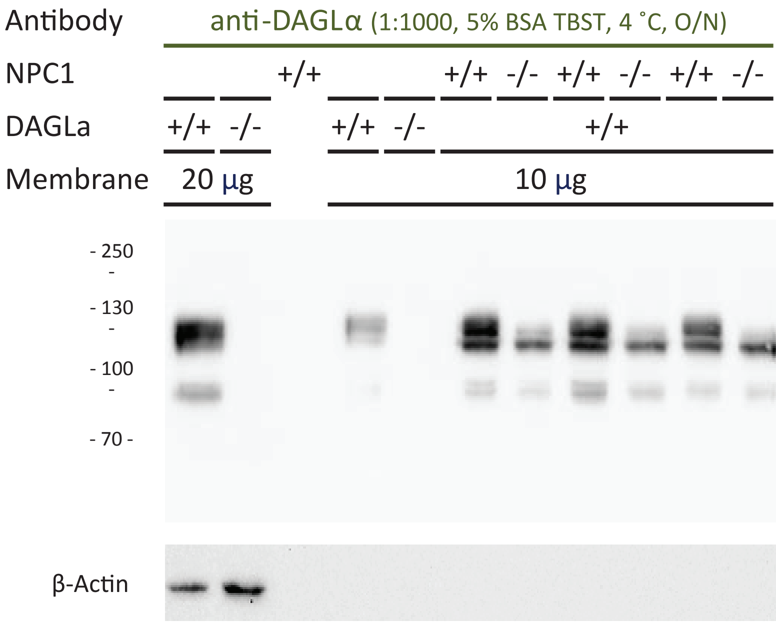
Antibody
anti-DAGLα (1:1000, 5% BSA TBST, 4 ˚C, O/N)
NPC1
+/+
+/+ -/- +/+ -/- +/+ -/-
DAGLa
+/+ -/-
+/+ -/-
+/+
Membrane
20 μg
10 μg
- 250
- 130
- 100
- 70 -
β-Actin
